# Transcription factor EHF drives cholangiocarcinoma development through transcriptional activation of glioma‐associated oncogene homolog 1 and chemokine CCL2

**DOI:** 10.1002/mco2.535

**Published:** 2024-05-13

**Authors:** Yiming Luo, Zhi Li, He Zhu, Junli Lu, Zhen Lei, Chen Su, Furong Liu, Hongwei Zhang, Qibo Huang, Shenqi Han, Dean Rao, Tiantian Wang, Xiaoping Chen, Hong Cao, Zhiwei Zhang, Wenjie Huang, Huifang Liang

**Affiliations:** ^1^ Hepatic Surgery Centre Tongji Hospital Tongji Medical College Huazhong University of Science and Technology Wuhan China; ^2^ State Key Laboratory of Biocatalysis and Enzyme Engineering School of Life Sciences Hubei University Wuhan China; ^3^ Key Laboratory of Breeding Biotechnology and Sustainable Aquaculture Institute of Hydrobiology Chinese Academy of Sciences Wuhan China; ^4^ Hubei Key Laboratory of Hepato‐Pancreato‐Biliary Diseases Wuhan China; ^5^ Key Laboratory of Organ Transplantation Ministry of Education, NHC Key Laboratory of Organ Transplantation, Key Laboratory of Organ Transplantation Chinese Academy of Medical Sciences Wuhan China

**Keywords:** cholangiocarcinogenesis, combined treatment, E26 transformation‐specific homologous factor, tumor‐associated macrophages

## Abstract

Cholangiocarcinoma (CCA) is characterized by rapid onset and high chance of metastasis. Therefore, identification of novel therapeutic targets is imperative. E26 transformation‐specific homologous factor (EHF), a member of the E26 transformation‐specific transcription factor family, plays a pivotal role in epithelial cell differentiation and cancer progression. However, its precise role in CCA remains unclear. In this study, through in vitro and in vivo experiments, we demonstrated that EHF plays a profound role in promoting CCA by transcriptional activation of glioma‐associated oncogene homolog 1 (GLI1). Moreover, EHF significantly recruited and activated tumor‐associated macrophages (TAMs) through the C‐C motif chemokine 2/C‐C chemokine receptor type 2 (CCL2/CCR2) axis, thereby remodeling the tumor microenvironment. In human CCA tissues, EHF expression was positively correlated with GLI1 and CCL2 expression, and patients with co‐expression of EHF/GLI1 or EHF/CCL2 had the most adverse prognosis. Furthermore, the combination of the GLI1 inhibitor, GANT58, and CCR2 inhibitor, INCB3344, substantially reduced the occurrence of EHF‐mediated CCA. In summary, our findings suggest that EHF is a potential prognostic biomarker for patients with CCA, while also advocating the therapeutic approach of combined targeting of GLI1 and CCL2/CCR2‐TAMs to inhibit EHF‐driven CCA development.

## Graphical abstract

EHF plays a crucial role in promoting cholangiocarcinoma (CCA) by activating GLI1 transcription. Additionally, EHF proficiently recruits and activates tumor‐associated macrophages via the CCL2/CCR2 axis, leading to tumor microenvironment remodeling. Furthermore, the strategic combination of GANT58 (a GLI1 inhibitor) and INCB3344 (a CCR2 inhibitor) significantly reduces EHF‐driven CCA occurrence.

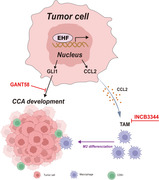

## 1 INTRODUCTION

Intrahepatic cholangiocarcinoma (CCA) accounts for 10%−15% of primary malignant liver cancers and its incidence has been increasing in recent years.^1^, ^2^, ^3^ Owing to its insidious onset, high risk metastasis, and the limited efficacy of various treatment modalities, CCA has a poor prognosis and high mortality rates.^4^ Postoperative adjuvant chemotherapy improves the survival of patients with CCA; however, drug resistance remains a challenge.^5^ Therefore, identifying new therapeutic targets and utilizing combination therapies are particularly important for improving the survival of patients with CCA.

ETS homologous factor (EHF), also known as epithelial‐specific ETS‐3, is a member of the E26 transformation‐specific (ETS) transcription factors family.^6^, ^7^ It selectively recognizes and binds to conserved sequences in the promoter region of target genes, which are rich in purines and have a GGAA/T core segment.^8^ It regulates several biological processes including cell migration, proliferation, differentiation, apoptosis, and angiogenesis.^9^ Using comparative transcriptome sequencing analysis of the transgenic zebrafish CCA model Tg (Lfabp10:Nras^Q61K^) and wild‐type zebrafish livers, we previously found that EHF was significantly overexpressed in CCA tissue that spontaneously formed in zebrafish.^10^ These studies suggested that EHF plays a critical role in the occurrence and development of cancer. However, the role of EHF in CCA remains unclear and clinical and translational studies on its oncogenic role are lacking.

Glioma‐associated oncogene homolog 1 (GLI1), a member of the zinc finger protein family, regulates cell proliferation, differentiation, and growth.^11^, ^12^ High levels of GLI1 expression have been associated with malignancy, prognosis, and treatment response in tumors.^13^ In tumor cells, GLI1 promotes cell proliferation, inhibits apoptosis, enhances cell migration and invasion, and participates in the occurrence and development of tumors by regulating the tumor microenvironment and inducing angiogenesis.^14^, ^15^, ^16^

The tumor microenvironment plays a pivotal role in the occurrence of CCA.^17^, ^18^ Tumor‐associated macrophages (TAMs), a crucial component of CCA inflammation, are recruited from circulating monocyte precursors by chemokines secreted by tumor cells, such as C‐C motif chemokine 2 (CCL2).^19^, ^20^, ^21^ TAMs promote tumor growth and development by producing various cytokines, including tumor necrosis factor‐alpha (TNF‐α), interleukin‐6 (IL‐6), and Vascular endothlial growth factor (VEGF).^22^, ^23^ Clinical evidence suggests that TAM infiltration is associated with poor prognosis in patients with CCA.^24^, ^25^ These studies highlighted the significant role of TAM infiltration in promoting CCA occurrence and metastasis.

In this study, we demonstrated for the first time that EHF promotes the proliferation of CCA cells and promotes macrophage infiltration by activating the transcription of GLI1 and CCL2. This activation led to the development of CCA. Moreover, combining the GLI1 inhibitor GANT58 with the C‐C chemokine receptor type 2 (CCR2) inhibitor INCB3344 significantly suppressed EHF‐induced CCA.

## 2 RESULTS

### 2.1 EHF is upregulated in human CCA and indicates poorer clinical outcomes

**FIGURE 1:**
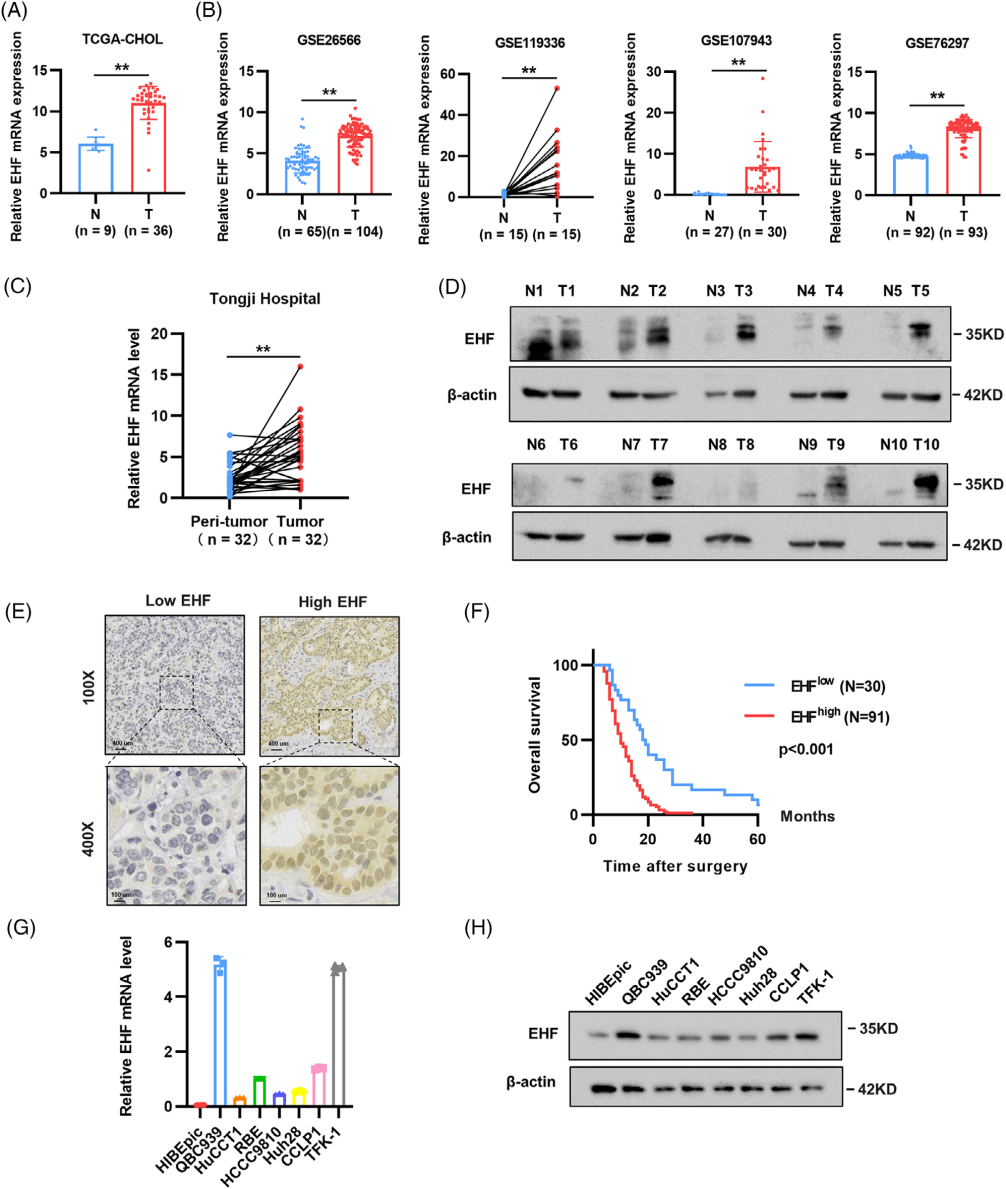
E26 transformation‐specific homologous factor (EHF) is upregulated in human cholangiocarcinoma (CCA) and indicates worse clinical outcomes. (A) EHF expression in normal tissues and CCA tissues from The Cancer Genome Atlas‐cholangiocarcinoma (TCGA‐CHOL) databases. (B) EHF expression in samples from the gene expression omnibus (GEO) database. (C and D) Relative expression of the EHF gene in 32 paired tumor and peri‐tumor tissues from Tongji cohort. (E) Representative images of immunohistochemistry (IHC) staining showing EHF protein expression in CCA tissues. Scale bars (up), 400 μm. (F) Kaplan–Meier plots of the overall survival (OS) rates of groups with differential EHF expression in Tongji cohort. (G and H) Relative mRNA and protein expression of EHF were shown in CCA cell lines and HIBEpiC cell line. *p < 0.05; **p < 0.01; ns: not significant. DEGs, differentially expressed genes; HIBEpiC, normal human intrahepatic biliary; N, normal; T, tumor.

Analyses conducted using the TCGA‐CHOL database revealed a significant upregulation of EHF transcript levels in CCA (Figure 1A). Accordingly, microarray data sets GSE26566, GSE119336, GSE107943, and GSE76297 from GEO database showed that EHF was overexpressed in CCA (Figure 1B). To validate the expression levels of EHF, we collected 32 pairs of CCA and adjacent normal tissues. Subsequent western blotting and real‐time qPCR analyses revealed a substantial upregulation of both EHF mRNA and protein levels in CCA compared to those in adjacent tissues (Figure 1C,D). Furthermore, we performed immunohistochemistry (IHC) staining to assess the expression of EHF in tumor tissues from 137 patients with CCA (Figure 1E). Based on the staining intensity, the patients were categorized into high and low expression groups, allowing us to investigate the correlation between EHF levels and patient clinical characteristics (Table S3). Statistical analysis demonstrated a positive correlation between EHF expression and tumor size as well as TNM stage (*p* < 0.05). Additionally, Kaplan–Meier analysis revealed that CCA patients with high EHF expression had poorer overall survival (OS) (*p* < 0.003) (Figure 1F). Subsequently, we examined the expression of EHF in normal bile duct and CCA cell lines and noted higher EHF protein and mRNA levels in CCA cells than in normal bile duct cell lines (Figure 1G,H). These findings suggest that EHF may serve as a prognostic indicator in CCA.

### 2.2 EHF promotes proliferation and growth of CCA cell in vitro

**FIGURE 2:**
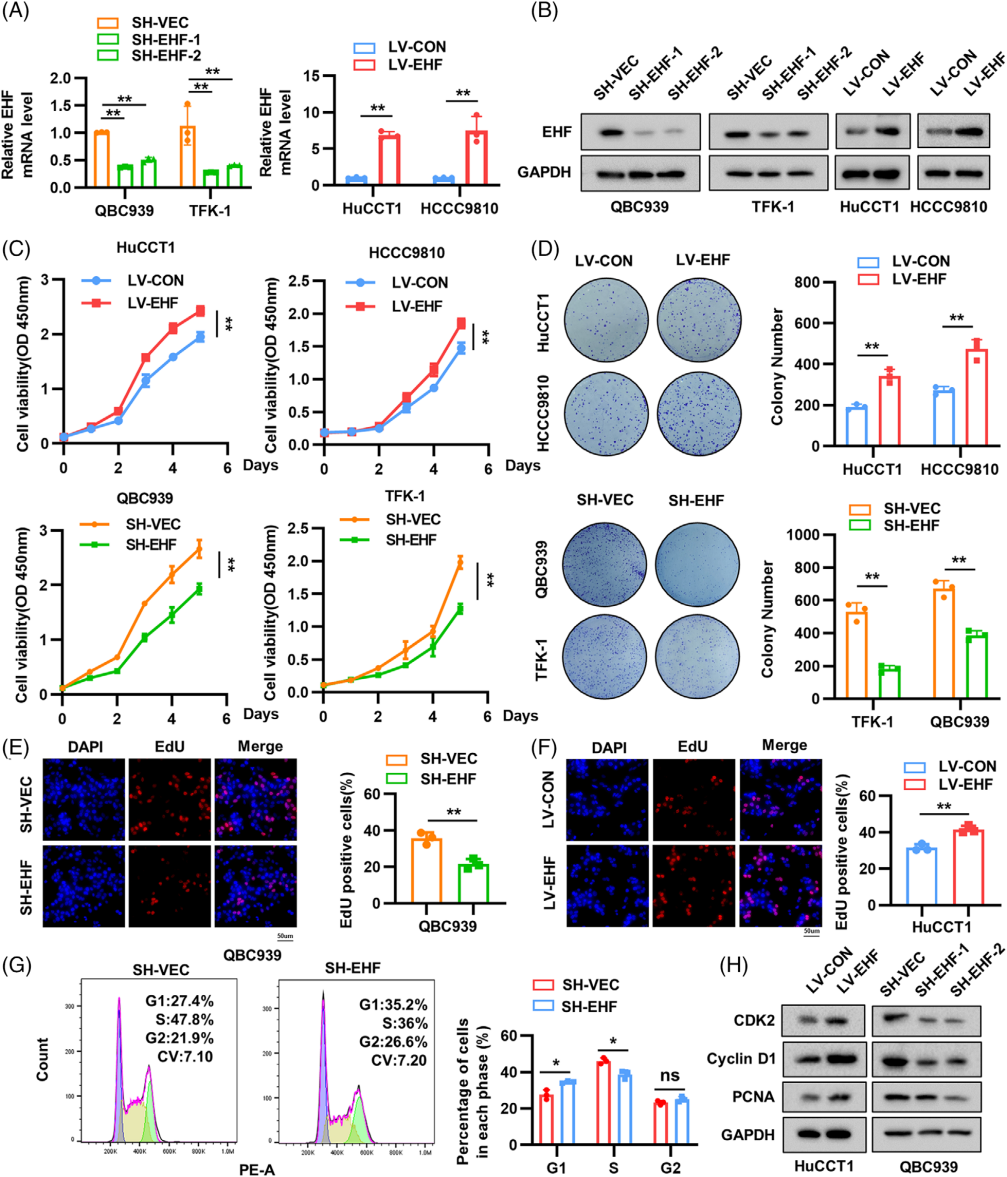
E26 transformation‐specific homologous factor (EHF) promotes cholangiocarcinoma (CCA) proliferation and growth in vitro. (A and B) Real‐time PCR and western blotting assays of EHF in the indicated cells transfected with lentivirus. (C) Growth curves measured by performing cell counting kit‐8 (CCK‐8) assay (OD 450 nm). (D) Representative images of colony formation assays and colony counts. (E and F) 5‐Ethynyl‐2′deoxyuridine (EdU) staining assays of HuCCT1 and QBC939 cells. (G) Representative flow cytometry of cell‐cycle stages of QBC939 cells. (H) Changes in the protein levels of proliferation‐related gene in HuCCT1 and QBC939 cells. *p < 0.05, **p < 0.01, based on the Student's *t* test. DAPI,4′,6‐diamidino‐2‐phenylindole.

To further investigate the biological behavior of EHF in CCA and its role in tumor development, we stably overexpressed EHF in HuCCT1 and HCCC9810 cell lines with low expression of EHF using a lentivirus and stably knocked down EHF in QBC939 and TFK‐1 cell lines with high expression of EHF using short hairpin RNA (shRNA), with SH‐EHF‐1 showing better knockdown efficiency (Figure 2A,B). Cell counting kit‐8 (CCK‐8) and colony formation assays showed that EHF overexpression significantly promoted the proliferation of HuCCT1 and HCCC9810 cells, whereas EHF knockdown markedly inhibited the proliferation of QBC939 and TFK‐1 cells (Figure 2C,D). EdU assay confirmed these results (Figure 2E,F). Cell cycle analysis further revealed that downregulation of EHF in QBC939 cells resulted in an increase in G1 phase cells and a reduction in S phase cells, whereas no significant difference was observed in the G2 phase, suggesting possible G1/S phase arrest (Figure 2G). We also examined some markers closely related to tumor cell proliferation, and the results showed that EHF promoted their expression in CCA cells (Figure 2H).

### 2.3 EHF promotes occurrence and development of CCA in vivo

**FIGURE 3:**
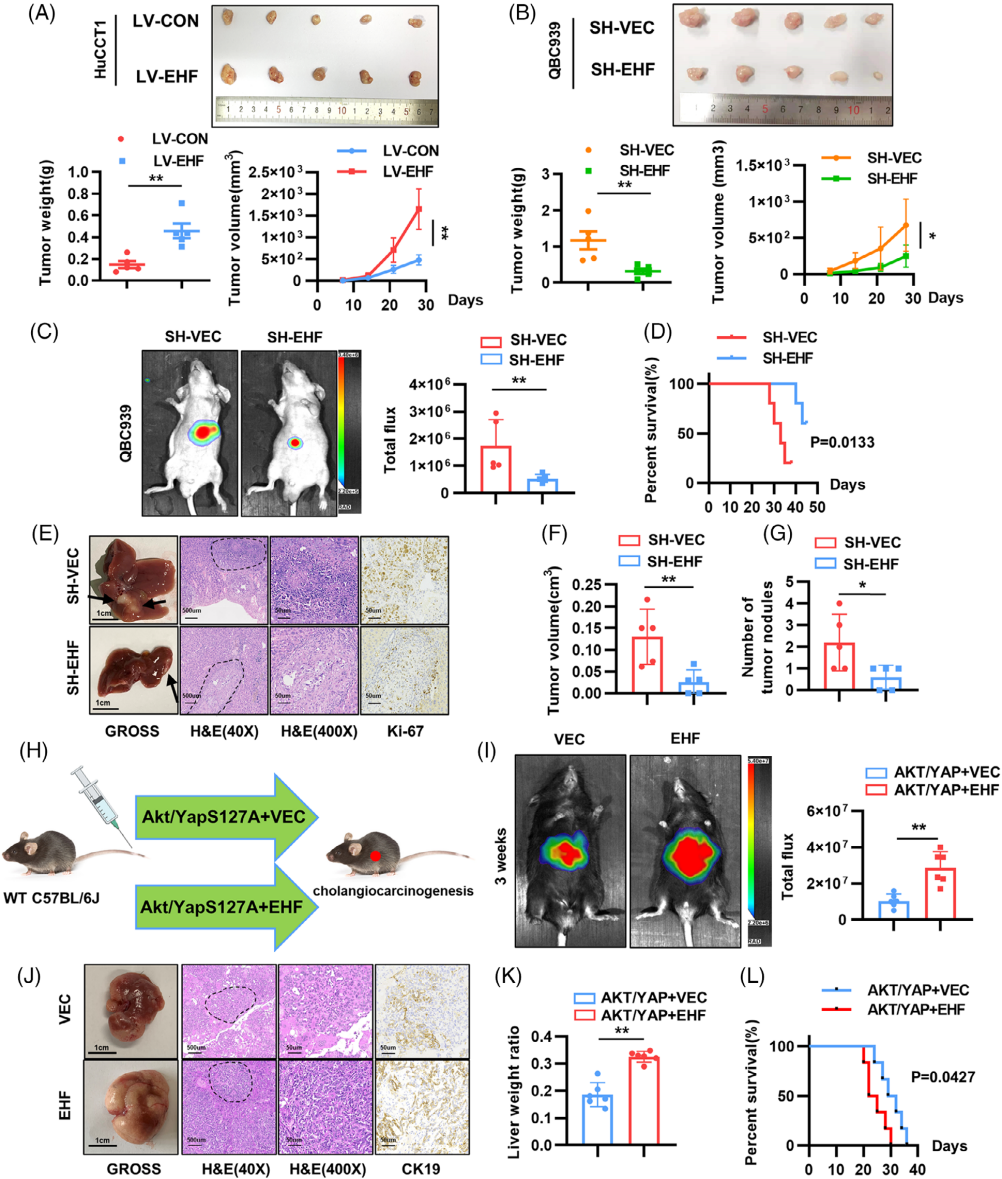
E26 transformation‐specific homologous factor (EHF) promotes cholangiocarcinoma (CCA) proliferation and growth in vivo. (A and B) Subcutaneous xenograft tumor volumes and weights measured for the different groups. (C) Representative bioluminescent pictures of nude mice performed orthotopic transplantation with QBC939 cells. (D) Overall survival in each group. (E) Representative pictures of gross, hematoxylin and eosin (H&E), and Ki‐67 staining images of the orthotopic CCA. Scale bars (gross), 1 cm. Scale bars (IHC), 50 μm. (F) Tumor volumes in the orthotopic model. (G) The number of visible tumor nodules. (H) Schematic diagram of hydrodynamic tail vein injection (HTVi) of oncogenic plasmids. (I) Representative bioluminescent pictures of AKT/Yap/vector and AKT/Yap/EHF mouse, respectively. (J) Representative pictures of Gross, H&E, and CK19 staining images. Scale bars (gross), 1 cm. Scale bars (IHC), 50 μm. (K) The ratio of tumor weight to body weight. (L) Kaplan–Meier plots of the overall survival (OS). *p < 0.05; **p < 0.01, based on the Student's *t* test.

We constructed subcutaneous xenograft, orthotopic CCA, and hydrodynamic tail vein injection (HTVi) models to investigate the effects of EHF in vivo. For the xenograft experiment, HuCCT1 and QBC939 cells were subcutaneously implanted into nude mice. The results showed that the volume and weight of tumors in the OE‐EHF group were higher than those in the control group, whereas tumors in the SH‐EHF group were smaller and lighter than those in the SH‐VEC group (Figure 3A,B). IHC also showed that the expression of Ki‐67, a marker of cell proliferation, was significantly increased in the LV‐EHF group and significantly decreased in the SH‐EHF group (Figure S1A). Next, orthotopic transplantation was performed by implanting fluorescent QBC939 cells into the livers of nude mice. The fluorescence intensity was significantly weaker in the SH‐EHF group than in the SH‐VEC group, and the survival time was significantly extended (Figure 3C,D). Gross images and HE staining results showed that the SH‐EHF group had a significant reduction in tumor size and number, and a significant reduction in KI‐67 staining intensity (Figure 3E–G). Finally, AKT (myr‐AKT) and YAP (YAP^S127A^) were transfected into mouse liver using a PT3 vector carrying a luciferase reporter gene to establish a mouse CCA model.^26^ The control group was injected PT3‐vec and the overexpression group was injected PT3‐EHF to observe the effect of EHF on CCA development (Figure 3H). The mice were sacrificed 3 weeks after injection. Gross images and histological analyses showed that the livers of the control mice had only small cystic nodules, whereas the livers of the LV‐EHF mice had significantly brighter fluorescence and heavier tumor burden than those of the control group mice, with clear solid tumors showing a ductal phenotype (Figure 3I–K). In addition, the survival time of mice in the LV‐EHF group was significantly shorter (Figure 3L). These results suggest that EHF promotes AKT/YAP‐driven CCA development. Based on these results, we conclude that EHF promotes the proliferation and development of CCA in vitro and in vivo.

### 2.4 GLI1 is transcriptionally activated by EHF in CCA

**FIGURE 4:**
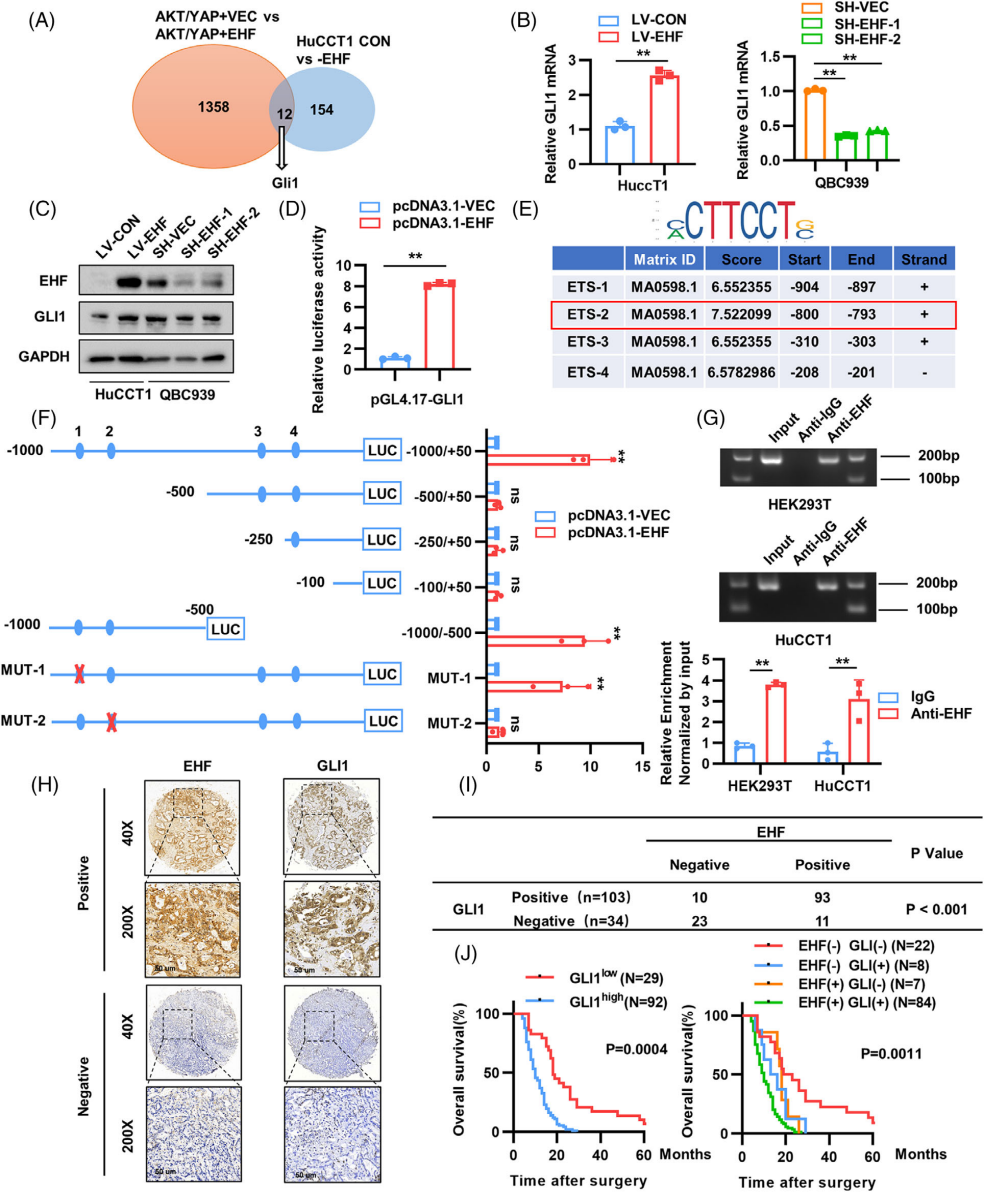
E26 transformation‐specific homologous factor (EHF) promotes cholangiocarcinoma (CCA) development through upregulating glioma‐associated oncogene homolog 1 (GLI1) expression in immunodeficient mice. (A) The diagram showed the genes regulated by EHF changes in mouse CCA model and CCA cell. (B and C) The mRNA and protein expression levels of GLI1 in indicated CCA cells. (D) Luciferase reporter assays of GLI1 promoter. (E) Schematic of the putative EHF binding motif and relative score determined using JASPAR. (F) Luciferase activity measured after the transfection of truncated and mutated GLI1 promote and pCDNA3.1‐EHF. (G) Chromatin immunoprecipitation (ChIP) assays revealed the interaction of EHF and GLI1 promoter in HEK293T and HuCCT1. (H) Representative immunohistochemistry (IHC) staining images of EHF and GLI1 in CCA cohorts. Scale bars, 50 μm. (I) The relevance of the expression of EHF and GLI1 in CCA cohorts. (J) Kaplan–Meier curves of the overall survival (OS) rates among groups with differential EHF/GLI1 expression in Tongji cohort.

To investigate the mechanism of EHF‐mediated CCA, we used RNA‐sequencing (RNA‐seq) to detect mRNA changes induced by EHF alterations in CCA cells and mouse CCA tissues. RNA‐seq analysis revealed that, compared to the control group, LV‐EHF overexpression led to significant upregulation of 154 genes in HuCCT1 cells, whereas the AKT/YAP/EHF group showed significant upregulation of 1358 genes in mouse CCA tissues compared to the AKT/YAP/PT3 group (Figure 4A). We further validated the 12 overlapping genes in CCA cells and found that interestingly, only mRNA expression of GLI1 decreased with EHF knockdown (Figure S1B). It has been reported that GLI1 is overexpressed in CCA and plays an important role in promoting CCA progression.^27^ Therefore, we explored whether GLI1 was involved in EHF‐mediated CCA occurrence.

Next, we validated the regulation of GLI1 expression by EHF in HuCCT1 and QBC939 cells. Overexpression of EHF upregulated the expression of GLI1, whereas EHF impaired the expression of GLI1 (Figure 4B,C). Considering that EHF is a transcription factor, we speculated that this regulatory effect occurs at the transcriptional level. We inserted the −1000 to +50 region of the GLI1 promoter into the PGL4.17 vector and found that luciferase activity was significantly enhanced after EHF transfection (Figure 4D). After obtaining the DNA binding sequence (ETS) of the EHF transcription factor from JASPAR, we predicted the promoter sequence of GLI1 and identified four potential DNA‐binding sites (Figure 4E). Based on this, we conducted a series of luciferase reporter gene experiments using truncation and point mutations in PGL4.17‐GLI1 (Figure 4F). Deletion of the segment between −1000 and −500 led to decreased activity of the GLI1 promoter. When the ETS‐2 binding site in this region was mutated, the activation of the GLI1 promoter by EHF transfection was also blocked, suggesting that ETS‐2 is likely the transcriptional binding site of EHF. In addition, chromatin immunoprecipitation (ChIP) and real‐time qPCR analyses showed that EHF effectively bound to the GLI1 promoter region in HEK293T and HuCCT1 cells (Figure 4G). Taken together, these results indicated that GLI1 is a direct transcriptional target of EHF.

We performed IHC staining on 137 CCA samples, and typical positive and negative images are shown in Figure 4H. Based on these results, we evaluated the potential correlation between EHF and GLI1 expression. The results showed that EHF expression was positively correlated with the expression of GLI1 (Figure 4I and Figure S1C), and Kaplan–Meier analysis showed that patients with positive GLI1 expression had a significantly lower OS rate (Figure 4J).

### 2.5 EHF promotes CCA proliferation and development by upregulating GLI1 expression in nude mice

**FIGURE 5:**
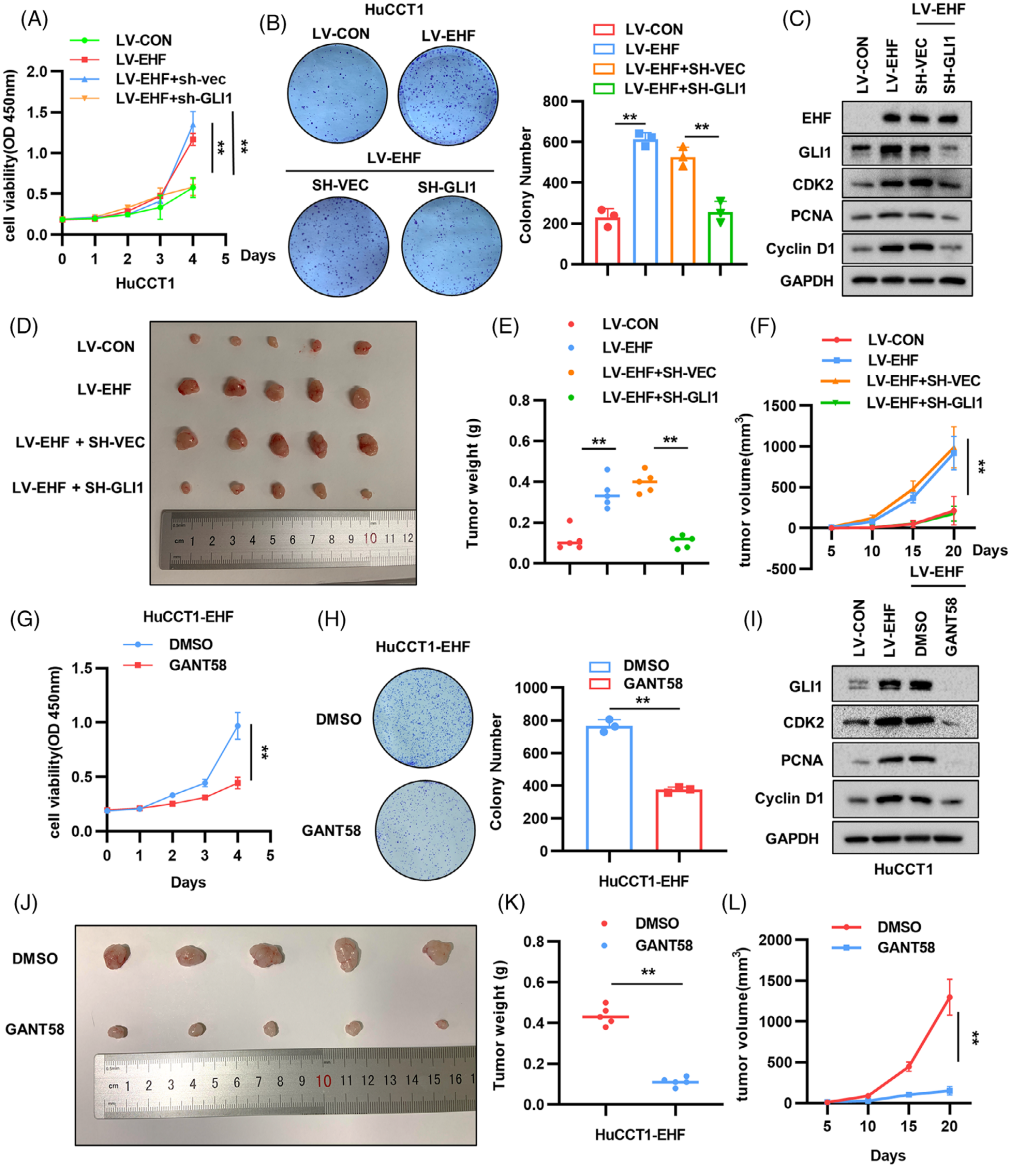
(A and B) Cell counting kit‐8 (CCK‐8) assays and colony formation assays of HuCCT1 cells with glioma‐associated oncogene homolog 1 (GLI1) knockdown on the basis of E26 transformation‐specific homologous factor (EHF) overexpression. (C) Expression levels of proliferation‐related genes in HuCCT1 cells based on EHF overexpression of GLI1 knockdown. (D–F) Gross images, tumor volumes, and weights after GLI1 knockdown on the base of EHF overexpression in the subcutaneous tumor model comprised of HuCCT1 cells. (G and H) The effect of GANT58 (10 μM) on cholangiocarcinoma (CCA) cell proliferation evaluated by CCK8 assay and colony formation assays. (I) Changes in the expression levels of proliferation‐related gene after using GANT58 (10 μM) in HuCCT1 cells. (J–L) Gross images, tumor weights, and volumes of the HuCCT1 xenografts treated with DMSO or GANT58 (25 mg/kg).

To validate the role of GLI1 in EHF‐induced CCA, we silenced GLI1 expression in HuCCT1‐LV‐EHF cells. CCK‐8 and colony formation assays showed that downregulation of GLI1 attenuated proliferation of HuCCT1 cells induced by EHF (Figure 5A,B). EHF overexpression significantly decreased the expression of the proliferation‐related genes (Figure 5C). Furthermore, we confirmed the role of GLI1 in EHF‐mediated CCA progression in nude mice, and found that the proliferative effect of EHF overexpression was indeed reversed by GLI1 knockdown (Figure 5D–F), and the relevant IHC results are shown in Figure S1D. We treated HuCCT1‐LV‐EHF cells with 10 μM GANT58, a specific antagonist of GLI1, and found that GANT58 significantly inhibited cell proliferation of HuCCT1 (*p* < 0.05) (Figure 5G,H). The expression of proliferation genes induced by EHF overexpression was also suppressed by GANT58 (Figure 5I). Similarly, the size and weight of subcutaneous xenograft tumors treated with GANT58 were significantly lower than those in the control group (Figure 5J–L).

### 2.6 Knockdown of GLI1 partially inhibits EHF‐promoted CCA development in immunocompetent mice

To make the study more relevant to humans, we constructed a polycistronic vector that carried the U6 promoter and shRNA targeting GLI1 in the PT3 vector expressing EHF, and then delivered the vector into target cells along with AKT/YAP, as shown in Figure S1E. Previously, we confirmed that EHF overexpression promoted CCA development in immunocompetent mice (Figure 3H–K). However, simultaneous knockdown of GLI1 only partially inhibited EHF‐induced CCA formation and prolonged the survival of mice to some extent (Figure S1F–I). Therefore, in immunocompetent animals, EHF may have regulatory mechanisms other than GLI1 that influence the occurrence and development of CCA.

### 2.7 EHF promotes CCA development by transactivating CCL2 expression

**FIGURE 6:**
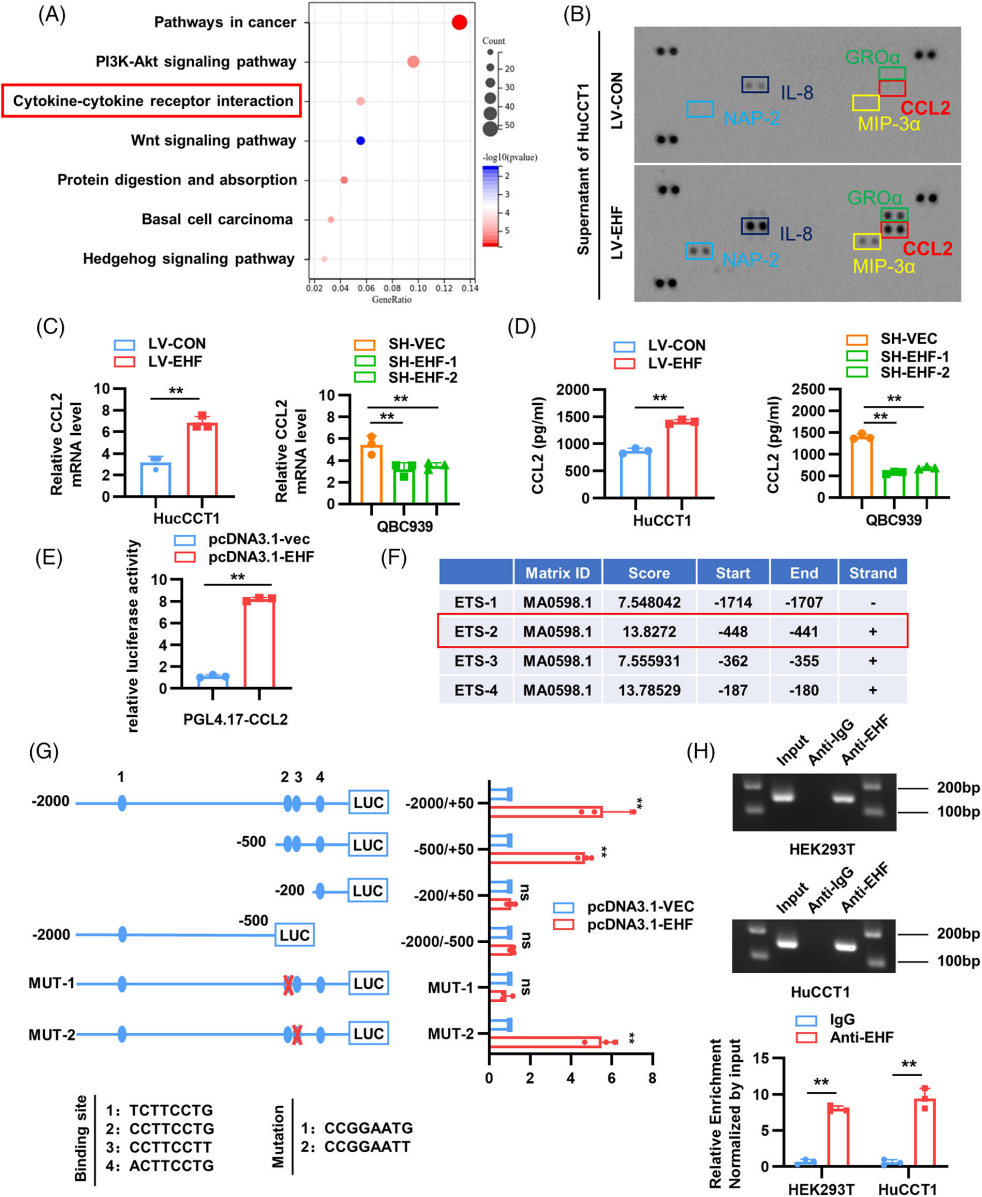
E26 transformation‐specific homologous factor (EHF) promotes the expression of C‐C motif chemokine 2 (CCL2) through transcription in cholangiocarcinoma (CCA). (A) KEGG analysis showing the significantly modulated signaling pathways after EHF overexpress in the AKT/YAP mouse. (B) Cytokine array was used to detect the difference of secreted factors in the supernatant of HuCCT1/LV‐CON and HuCCT1/LV‐EHF cells. (C and D) Real‐time PCR and ELISA showing the relative levels of CCL2 in indicated CCA cells. (E) Luciferase reporter assays of CCL2 promoter. (F) Relative score determined in CCL2 promotor using JASPAR. (G) Luciferase activity measured after the transfection of truncated and mutated CCL2 promote and pCDNA3.1‐EHF. (H) Chromatin immunoprecipitation (ChIP) assays revealed the interaction of EHF and CCL2 promoter in HEK293T and HuCCT1.

To explore additional carcinogenic mechanisms of EHF within the tumor immune microenvironment, we conducted a Kyoto Encyclopedia of Genes and Genomes (KEGG) enrichment analysis of the sequencing results of mouse CCA tissues. Interestingly, we discovered significant upregulation of the cell–cell signaling pathway in the EHF overexpression group (Figure 6A). Previous research has shown that cytokines can promote CCA progression by attracting immunosuppressive cells, such as TAMs and MDSCs, to the tumor microenvironment,^28^, ^29^ suggesting that EHF may affect CCA progression through cell–cell interactions. To investigate the mechanism by which cytokines or chemokines are involved in EHF‐mediated CCA occurrence, we used a cytokine array to measure the secretion of cytokines from HuCCT1 cells overexpressing EHF (HuCCT1 LV‐EHF) compared to the control group (HuCCT1 LV‐CON) (Figure 5B). The results revealed a significant increase of CCL2, NAP2, GROα, among others in the supernatant of HuCCT1 LV‐EHF cells, with CCL2 exhibiting the highest increase (Figure S2A). CCL2, also known as MCP‐1, interacts with the CCR to recruit TAMs.^30^ Next, we determined whether EHF regulates the expression of CCL2. Real‐time PCR and Enzyme linked immunosorbent assay (ELISA) experiments showed that EHF overexpression increased the expression and secretion of CCL2 in HuCCT1 cells, whereas EHF knockdown decreased the expression and secretion of CCL2 in QBC939 cells (Figure 6C,D). Luciferase reporter gene experiments showed that EHF activated the CCL2 promoter (Figure 6E). Truncation and point mutations of the CCL2 promoter at the four predicted EHF‐binding sites revealed that ETS‐2 was crucial for EHF‐induced CCL2 transactivation (Figure 6F,G). ChIP experiments further confirmed that EHF directly binds to the CCL2 promoter (Figure 6H). These results indicate that CCL2 is also a direct transcriptional target of EHF.

### 2.8 EHF promotes CCA development by CCL2/CCR2 pathway‐induced TAM infiltration and remodeling of the tumor microenvironment

**FIGURE 7:**
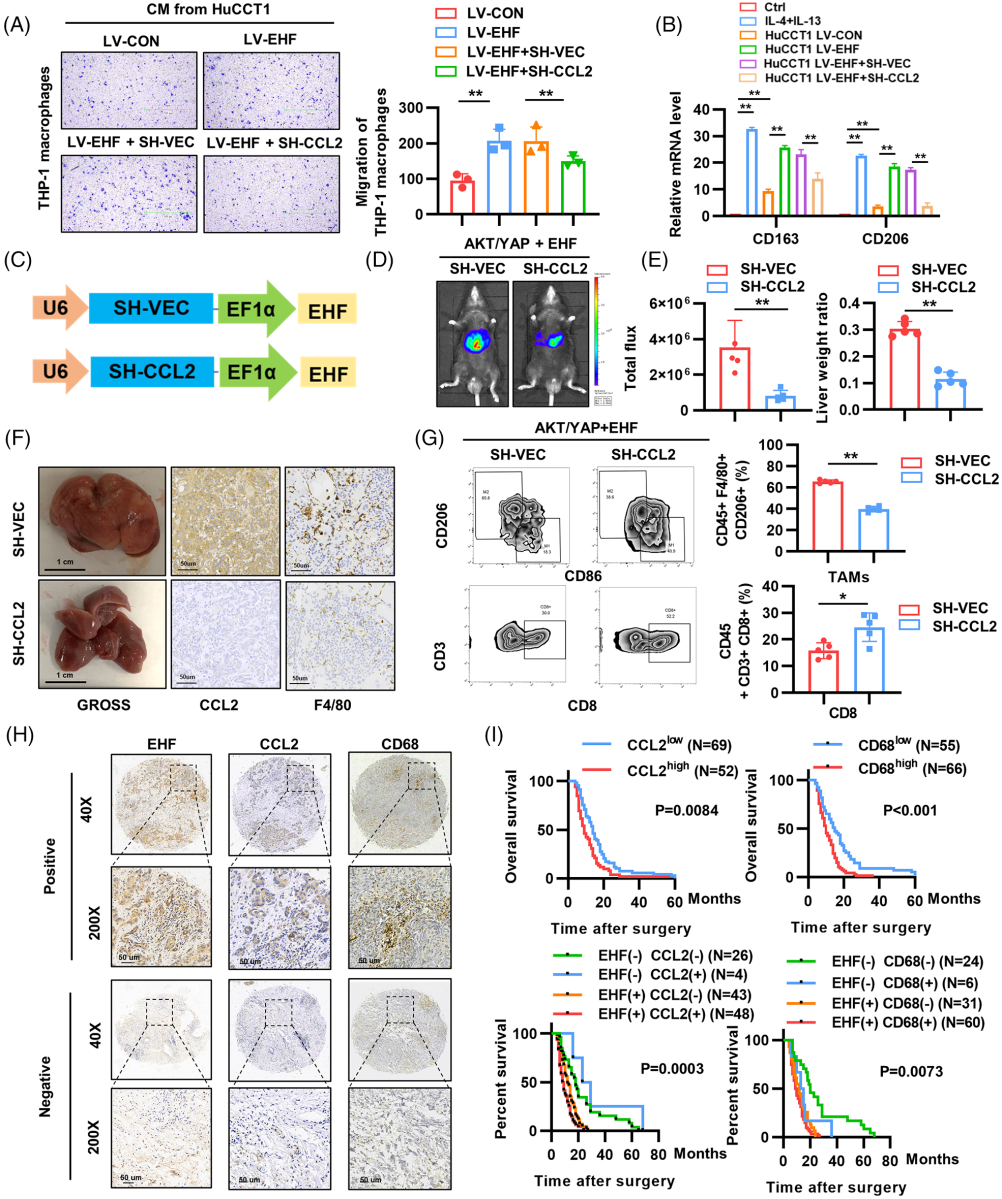
E26 transformation‐specific homologous factor (EHF) promotes cholangiocarcinoma (CCA) development through C‐C motif chemokine 2/C‐C chemokine receptor type 2 (CCL2/CCR2) pathway‐induced M2 macrophage infiltration. (A) Chemotaxis assays reflected the recruitment effect of CMs of HuCCT1 on THP1 macrophages. (B)The mRNA levels of CD163 and CD206 in THP‐1 macrophages cultured with CMs of HuCCT1 cells. (C)Schematic diagram of constructing plasmid of SH‐VEC and SH‐CCL2. (D) Representative bioluminescent pictures of AKT/Yap/EHF+ SH‐VEC and SH‐CCL2 mouse, respectively. (E) The ratio of tumor weight to body weight. (F) Representative pictures of gross, CCL2, and F4/80 staining images. Scale bars (GROSS), 1 cm. Scale bars (immunohistochemistry [IHC]), 50 μm. (G) Percentages of tumor‐associated macrophages (TAMs) and CD8^+^ T cells counted by Flow Cytometry (FCM). (H) Representative IHC staining images of EHF, CCL2, and CD68 in CCA cohort. Scale bars, 50 μm. (I) The relevance of the expression of EHF and CCL2 or CD68 in CCA cohorts. (J) Kaplan–Meier curves of the overall survival (OS) rates among groups with differential EHF/CCL2 or CD68 expression in Tongji CCA cohort.

Previous studies have reported that CCL2 binds mainly to its receptor, CCR2, to recruit and activate TAMs, which invade tumor sites and promote tumor progression.^20^, ^31^, ^32^ Therefore, we investigated whether CCL2 is involved in EHF‐induced CCA through the recruitment and activation of TAMs. First, to observe the effect of EHF on macrophages, we used 8‐μm‐transwell chambers to connect the conditioned medium from CCA cells and macrophages (Figure S2B). Chemotaxis assays demonstrated that the supernatant from the HuCCT1‐EHF group significantly increased the migratory ability of THP‐1 macrophages compared to that of the HuCCT1‐CON group. Additionally, simultaneous knockdown of CCL2 significantly reduced the migratory ability of THP‐1 macrophages induced by EHF overexpression (Figure 7A). Furthermore, treatment with the CCR2 antagonist INCB3344 significantly inhibited the enhanced migratory ability of macrophages (Figure S2C). Moreover, we observed that the conditioned medium from HuCCT1‐EHF cells affected the polarization of THP1 macrophages. After co‐culture, M2 macrophage markers such as CD163, CD206, IL‐10, and TGFB‐1 were significantly upregulated, and knockdown of CCL2 attenuated the upregulation of M2 macrophage markers (Figure 7B and Figure S2D). Based on the AKT/YAP‐induced HTVi CCA model, we constructed and injected plasmids containing PT3‐EHF‐SH‐CCL2, a polycistronic vector targeting CCL2 (Figure 7C). The results showed that bioluminescence and tumor burden in the livers of mice in the SH‐CCL2 group were significantly lower than those in the SH‐VEC group, and the number of F4/80‐labeled macrophages was also significantly reduced (Figure 7D–F). In addition to the reduced total number of recruited macrophages, we found that the proportion of M2 macrophages decreased significantly, whereas the proportion of CD8^+^ T cells increased in the SH‐CCL2 group using flow cytometry (Figure 7G).

We used clodronate liposomes to deplete macrophages in mice to determine whether macrophage infiltration is involved in EHF‐mediated cholangiocarcinogenesis. Bioluminescence and gross liver results showed that CCA development was significantly slower in the macrophage‐depleted group, whereas the survival time of the mice was prolonged (Figure S2E–H). These results suggest that EHF‐mediated macrophage infiltration promotes CCA development.

Immunohistochemical staining of EHF and CCL2 expression in patients with CCA, as well as macrophage infiltration (CD68 as a marker), revealed a positive correlation between EHF expression levels and CCL2 and CD68 expression levels (Figure 7H,I and Figure S2I–K). In the CCA cohort, CCL2‐positive expression and accumulation of TAMs, as indicated by CD68‐positive expression, were both associated with poor prognosis, and patients with EHF(+)/CCL2(+) or EHF(+)/CD68(+) had the worst prognosis (Figure 7J). In conclusion, these results suggest that in CCA, EHF can promote the recruitment of macrophages and their polarization toward the M2 phenotype by transcriptionally regulating CCL2, resulting in the progression of CCA.

### 2.9 Combined treatment with the GLI1 inhibitor GANT58 and the CCR2 inhibitor INCB3344 significantly reduces EHF‐driven cholangiocarcinogenesis

**FIGURE 8:**
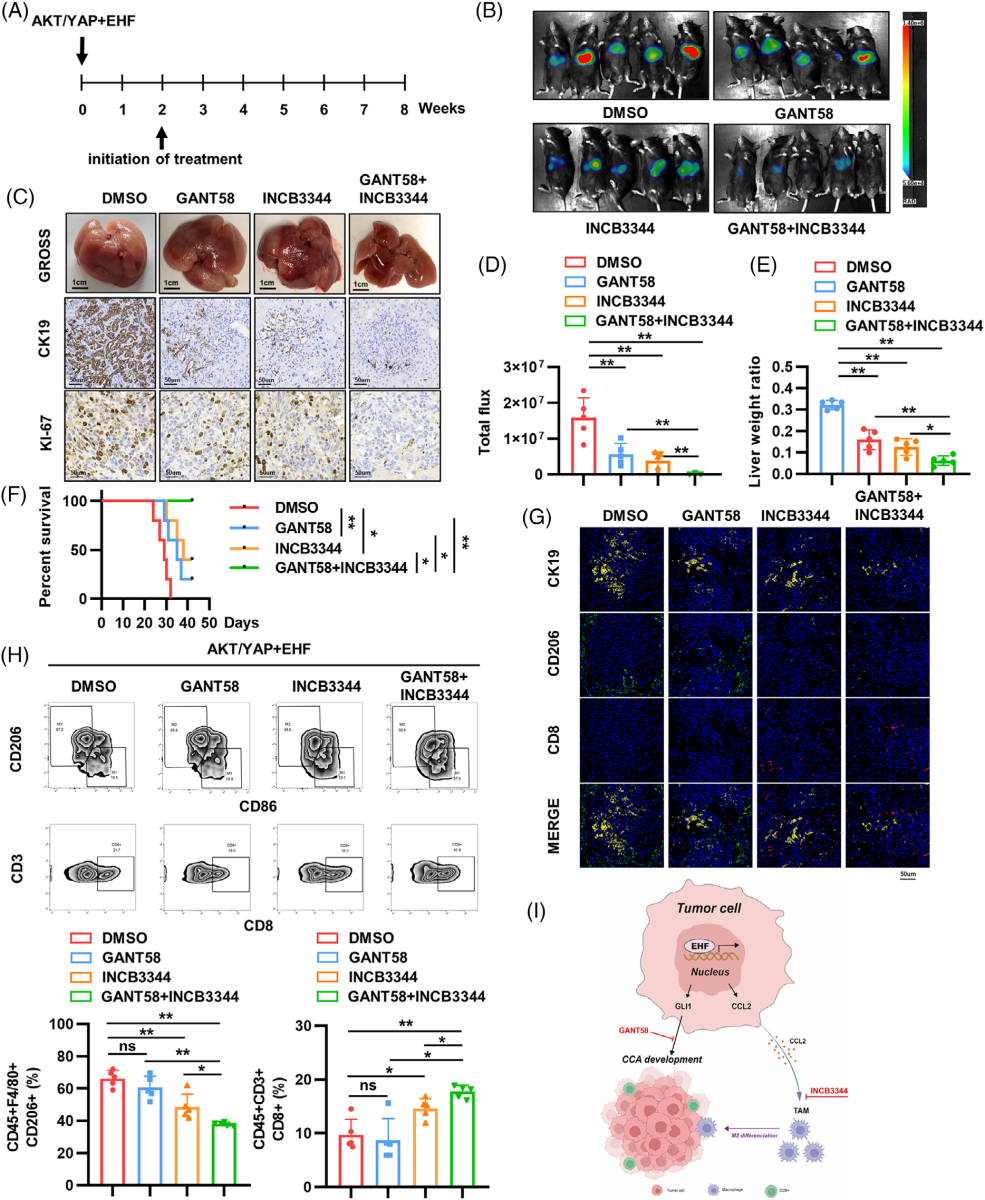
Combined treatment of glioma‐associated oncogene homolog 1 (GLI1) inhibitor GANT58 and CCL2/CCR2 inhibitor INCB3344 dramatically decreased E26 transformation‐specific homologous factor (EHF)‐driven cholangiocarcinoma (CCA) development. (A) The diagram of combined treatment in C57/BL mice. (B–E) In vivo assays showed that combined treatment of GLI1 and CCR2 inhibitors can almost block CCA development totally. (B) Representative bioluminescence images of different groups. (C) Representative gross, CK19, and Ki‐67 staining images. Scale bars (gross), 1 cm. Scale bars (immunohistochemistry [IHC]), 50 μm. (D) Analysis of the intensity of mouse liver bioluminescence. (E) The ratio of tumor weight to body weight in different treatment groups. (F) Survival curves of mice in different treatment groups. (G) Multi‐color immunofluorescence (mIF) showed the infiltration of CCA epithelia (CK19^+^), tumor‐associated macrophages (TAMs) (CD206^+^), and CD8^+^ T cells (CD8^+^) in different groups. Scale bars, 50 μm. (H) Flow cytometry showed the percentage of TAMs (CD206^+^) and CD8^+^ T cells. (I) A schematic diagram of the function of the EHF‐GLI1/CCL2‐macrophage pathway in CCA development. *p < 0.05, **p < 0.01.

Our previous study demonstrated that EHF promotes cholangiocarcinogenesis through the transcriptional activation of GLI1 and CCL2 expression. Thus, we used a combination of a GLI1‐specific small‐molecule inhibitor, GANT58, and a CCL2/CCR2 inhibitor, INCB3344, to observe their effects on EHF‐induced CCA occurrence. We used a mouse model of CCA induced by HTVi as the animal experimental model. Two weeks after plasmid injection, the mice were treated with GANT58 and/or INCB3344 for 3 weeks (Figure 8A). The drugs were previously validated, and our results showed that the combination treatment had no significant toxicity in mice (Figure S3A–D). The combined treatment with GANT58 and INCB3344 significantly reduced the intensity of liver luciferase, reduced the liver tumor burden, and prolonged the OS of mice compared to the control group or monotherapy (Figure 8B–F). Multicolor immunofluorescence (mIF) and flow cytometry analysis of tumor tissues demonstrated that the infiltration of M2 macrophages (CD206^+^) was significantly decreased, whereas that of CD8^+^ T cells was significantly increased in the combination treatment group, indicating an improvement in the immunosuppressive tumor microenvironment (Figure 8G,H). These studies suggest that the combination of the GLI1 inhibitor GANT58 and the CCL2/CCR2 inhibitor INCB3344 effectively inhibited EHF‐induced CCA occurrence (Figure 8I).

## 3 DISCUSSION

In our previous study, we found that the transcription factor EHF was highly expressed in a zebrafish CCA model.^10^ In recent years, dysregulation of EHF expression has been reported in various cancers; however, its specific mechanisms in different types of cancers have been inconsistent.^33^, ^34^ This is mainly because the ETS domain can act as both a suppressor and promoter of the occurrence and development of cancer depending on the specific type of cancer.^35^ For example, in patients with prostate cancer, low EHF expression promoted epithelial‐mesenchymal transition ultimately leading to carcinogenesis and cancer cell metastasis.^36^ Similarly, in pancreatic cancer, EHF inhibited stemness of pancreatic cancer by downregulating CXCR4.^37^ In contrast, high EHF expression in patients with ovarian cancer was associated with poor prognosis, and the inhibition of EHF levels significantly reduces the metastatic ability of ovarian cancer cells in vitro and in vivo.^38^, ^39^ In colon cancer cell lines, research has shown that EHF directly activated RUVBL1 and inhibited apoptosis induced by p53, thereby promoting cell proliferation.^40^ In this study, we discovered that the expression level of EHF was significantly higher in cancer tissues of patients with CCA than in adjacent noncancerous tissues, and that it was correlated with tumor size and TNM staging. Furthermore, in in vitro and animal experiments, we observed that EHF promoted the proliferation of CCA cells and cholangiocarcinogenesis, and that the oncogenic effect was weakened when EHF was inhibited. These results suggested that EHF plays an important role in the occurrence and development of CCA.

GLI1, a key member of the Hedgehog (Hh) signaling pathway, plays a pivotal role in tumorigenesis.^41^ Aberrant activation of the Hh signaling pathway has been shown to positively regulate tumorigenesis in human cancers, including CCA.^42^, ^43^ Inhibitors of the Hh pathway also been reported to suppress the development of CCA.^44^ After collecting the supernatant from CCA cells and exploring the changes in its composition using a cytokine array, we observed that the regulation of CCL2 by EHF was particularly significant. CCL2, also known as MCP‐1, recruits macrophages from the bloodstream to the microenvironment by binding to CCR2 on the macrophages.^20^, ^45^, ^46^ Previous studies have reported that treatments targeting CCL2‐mediated recruitment of TAMs in tumors have also shown some efficacy.^31^, ^47^ Upon validating this hypothesis and using a macrophage‐depleting agent, we observed a significant inhibition of the pro‐carcinogenic effect of EHF, indicating a crucial role of macrophages in the pro‐carcinogenic mechanism of EHF. These studies indicated that GLI1 and CCL2 play important roles in CCA development.

In this study, we found that EHF directly binds to the promoter regions of GLI1 and CCL2, thereby promoting their transcription, as demonstrated by dual‐luciferase reporter gene assays and ChIP experiments. In addition, GLI1 blockade effectively attenuated EHF upregulation in tumor cells and immunosuppressed animal models, thereby reducing CCA cell proliferation and growth. Interestingly, this inhibitory effect was incomplete in the immunocompetent mice. We speculate that this may be due to the fact that the oncogenic effect of GLI1 mainly occurs within tumor cells, and there may be mechanisms of intercellular interactions when studying the tumor microenvironment. As a potent oncogenic transcription factor, EHF is likely to exert its effects through multiple mechanisms not limited to a single pathway. Other important target genes may also play a role in the interaction between tumor cells and other cells. Therefore, by analyzing the tissue sequencing results of an immunocompetent mouse model, we found that the cytokine pathway plays a key role and identified CCL2 as a key effector gene. We validated that EHF promotes the migration and infiltration of TAMs into tumors through the CCL2/CCR2 axis, which complements the function of GLI1 in tumor cells and provides a more comprehensive mechanism for EHF‐mediated cholangiocarcinogenesis. Regarding the impact of EHF on other immune cells such as T cells, we speculate that it primarily exerts its effects indirectly by influencing macrophages. Validation by flow cytometry revealed that the regulation of T cells varied with the level of macrophage infiltration, which is consistent with the reported immunosuppressive functions of TAMs.

However, it must be acknowledged that our research has certain limitations. While the hydrodynamic mouse model we utilized can reflect the in vivo occurrence of CCA to some degree, it is undeniable that the inclusion of transgenic mice in experiments may provide stronger support for our findings. Nevertheless, we have conducted thorough deliberations in both in vivo and in vitro settings, across various dimensions, to mitigate this limitation to a certain extent.

The significance of targeted therapy for CCA lies in the development of a treatment strategy that specifically targets gene abnormalities, effectively prolongs patient survival, and improves treatment outcomes. Based on the important role of EHF in CCA, we blocked the development of EHF‐induced CCA by targeting GLI1 and CCL2. GANT58 is a small‐molecule inhibitor specific to GLI1, which has significant inhibitory effects on tumor cells in vitro but lacks in vivo and clinical trials. INCB3344 is an effective and specific CCL2/CCR2 antagonist that reduces the number of macrophages in target tissues. Our animal experimental results showed that combined treatment with these two inhibitors significantly inhibited EHF‐mediated CCA development compared to the control or single‐drug treatment. These results suggest a novel strategy for the inhibition of EHF‐induced cholangiocarcinogenesis.

## 4 CONCLUSIONS

In conclusion, our research results suggest that EHF is an oncogenic gene that promotes CCA development through both immune and non‐immune pathways. In the non‐immune pathway, EHF promotes the expression of GLI1, while in the immune pathway, EHF promotes CCL2 secretion to recruit and polarize macrophages. Combined targeted therapy against GLI1 and CCL2/CCR2‐TAMs can significantly inhibit EHF‐mediated CCA development, providing a promising treatment strategy for CCA patients with EHF upregulation.

## 5 MATERIALS AND METHODS

### 5.1 Clinical samples

This study was approved by the Ethics Committee of Tongji Medical College. Tumor and paired tissues adjacent to the carcinoma were collected from 32 patients diagnosed with CCA who underwent clinical surgery at the Hepatic Surgery Center, Tongji Hospital (Wuhan, China) between 2014 and 2015. Furthermore, additional 137 cases of clinical and pathological follow‐up data for patients with CCA (between 2013 and 2015) were provided by the Hepatic Surgery Center, Tongji Hospital (Wuhan, China). All patients, who provided informed consent, were followed for up to 65 months.

### 5.2 Animal studies

The Institutional Animal Care and Treatment Committee of the Huazhong University of Science and Technology granted ethical approval for the animal experiments. Four‐week‐old male BALB/c nude mice and C57BL/6J mice were obtained from Hubei Biont Biological Technology Co., Ltd. The mice were randomly divided into groups of five per cage. For the subcutaneous graft model, 5 × 106 HuCCT1 or QBC939 cells were resuscitated with 100 μL RPMI medium and injected into the armpit of each nude mouse. After 4 weeks, the mice were sacrificed and the weight and volume of the tumors were recorded. Tumor volume was calculated using the following formula: volume  = (length × width)2/2. Following the experiment, specimens were fixed with 4% formaldehyde. In the intrahepatic tumor implantation model, 2 × 10^6^ QBC939 cells were resuspended in a mixture of 30 μL RPMI medium and Matrigel, then inoculated under the left lobe capsule of the liver. Mice were euthanized 6 weeks after inoculation or when they became severely weakened, and their livers were collected for follow‐up experiments.

In the HTVi model, 20 μg myr‐AKT, 30 μg YAPS127A, and 2.75 μg SB100 plasmids were co‐injected to trigger spontaneous CCA development in C57BL/6J mice. For combination therapy in animals, following plasmid injection, mice received intraperitoneal injections of GANT58 (25 mg/kg) (MedChemExpress) and/or INCB3344 (30 mg/kg) (Selleck) every other day, starting 2 weeks after the injection. Upon completion of the experiments, the mice were euthanized and their livers were harvested for flow cytometry and IHC staining.

### 5.3 RNA extraction and quantitative RT‐PCR

Total RNA was extracted from patient tissues and cell lines using a FastPure Cell/Tissue Total RNA Isolation Kit V2(Vazyme Biotech Co., Ltd) according to the manufacturer's protocol. Then, 2 μg of isolated RNA was transcribed to cDNA using the HiScript III Q Select RT SuperMix for qPCR (Vazyme Biotech Co., Ltd). Quantitative RT‐PCR was performed using 2 xQ3 SYBR qPCR Master Mix (Universal) (#22204, Tolo Biotech Co., Ltd). The primer sequences are presented in Table S2. The internal gene GAPDH was used as a normalized control for the qRT‐PCR assay, and the data were based on at least three independent experiments.

### 5.4 Western blot analysis

Cells or tissues were lysed with RIPA lysis buffer at 4°C for 20 min. Protein samples were quantified using a BCA protein assay kit (Thermo Fisher Scientific). Proteins were separated by SDS‐PAGE (Yeasen) and transferred onto PVDF membranes (Millipore). The electrophoresis solution is configured with tris‐HCI, glycine, and SDS (Beijing Dingguo changsheng Biotechnology Co., Ltd). The membranes were then blocked with 5% bovine serum albumin (BSA) at 37°C for 60 min, and incubated with primary antibodies overnight at 4°C. The following day, the membranes were incubated with specific horseradish peroxidase (HRP)‐conjugated secondary antibodies at 37°C for 1 h. Finally, the proteins were visualized using an enhanced chemiluminescence (ECL) detection system (Bio‐Rad). Primary and secondary antibodies used are listed in Table S1.

### 5.5 Immunohistochemistry staining

The samples were fixed in 4% paraformaldehyde for at least 24 h and embedded in paraffin. Note that 4‐μm thick sections were cut and stained with hematoxylin and eosin for histological analysis (Biossci). Paraffin sections were heated to 65°C for 30 min and dewaxed with xylene and alcohol of different concentrations. The slices were then heated to 95°C for 30 min for antigen repair, and then soaked in 3% H_2_O_2_ for 10 min after cooling. BSA (5%) was closed at 37°C for 60 min and incubated with primary antibody at 4°C overnight. On the second day, the sections were incubated with HRP conjugate secondary antibodies for 1 h. Finally, the peroxidase substrate solution 3,3'‐diaminobenzidine (DAB, Vectorlabs) was dripped until the desired staining strength was achieved. The sections were stained with hematoxylin, dehydrated, sealed, observed, and stained under a microscope. Immunohistochemical stain scoring of human CCA samples was performed by two pathologists in a double‐blind manner. In each case, five microscopic fields were examined, and scores were assigned based on the percentage of positively stained cells and staining intensity. If less than 5% of the cells were stained, a score of 0 was assigned. Scores of 1, 2, and 3 were assigned for staining in 26%−0%, 51%−75%, and 76%−100% of the cells, respectively. The staining intensity was assessed on a scale of 0 (colorless) to 3 (dark brown). The final staining score was the product of two individual scores.

### 5.6 Cell culture

Human normal bile duct cell line and CCA cell lines HuCCT1, RBE, QBC939, TFK‐1, HCCC9810, Huh‐28, and CCLP1 were purchased from American Type Culture Collection (ATCC) and were cultured in RPMI‐1640 medium (Pricella Life Science & Technology Co., Ltd) supplemented with 10% fetal bovine serum (Scitecher, Beijing Dingguo Changsheng Biotechnology Co., Ltd) at 37°C in a 5% CO_2_ incubator.

### 5.7 Construction of lentivirus and stable cell lines

A lentiviral system was used to construct cell lines with stable knockdown and overexpression. shRNAs targeting EHF, GLI1, and CCL2 were inserted into the PLKO.1 vector (Invitrogen) to construct knockdown plasmids. Plasmids was extracted by using a Steadypure Plasmid DNA Extraction Kit (Accurate Biotechnology (Hunan)Co., Ltd) following the manufacturer's instructions. A non‐specific scramble shRNA sequence was used as negative control. The shRNA sequences can be found in Table S2. EHF was also inserted into the plenti vector (Invitrogen) to construct overexpression plasmids. All the above plasmids were confirmed by DNA sequencing. Lentivirus‐infected stable cell lines were screened with puromycin (5 μg/mL) for 2 days.

### 5.8 CCK8 assay

Cells were seeded onto 96‐well plates at a density of 100 cells/well. Each sample was treated with 100 μL CCK8 solution, which was diluted 10 times with RPMI medium, and incubated at 37°C for 1 h. Absorbance values were measured using a spectrophotometer.

### 5.9 Colony formation assay

CCA cells were seeded at a density of 1000 cells per well in six‐well plates and cultured for a duration of 2 weeks. Following this, the cells were fixed with 4% formaldehyde and stained with 0.1% crystal violet for 15 min. Finally, the colonies were counted.

### 5.10 EdU assay

Cells were seeded onto 24‐well plates at a density of 5.0 × 105 cells/well. To each well, 10 μM EdU was added and the cells were incubated at 37°C for 1 h. The supernatant was then removed and the cells were fixed with 4% formaldehyde for 30 min. After being washed twice with PBS, the cells were incubated with 0.3% Triton X‐100 for 10 min. The Apollo was added to the cultured cells, which were then incubated in the dark at room temperature for 30 min. The cell nuclei were stained with DAPI for 10 min, and images were obtained under a microscope.

### 5.11 Cell cycle analysis

After achieving 80% confluence during cell culture, the cells were harvested and resuspended into a single‐cell suspension. These cells were then fixed using 70% ethanol and stored at 4°C overnight. Following this, the ethanol was removed, and PI staining was performed by incubating the cells at room temperature and in the dark for 15 min. Finally, the cells were detected by flow cytometry.

### 5.12 RNA‐sequencing

Total RNA from HTVi‐induced CCA mice and HuCCT1 cells was extracted for RNA sequencing. Sequencing and data analysis services were provided by Wuhan Generead Biotechnologies Co. Ltd. To identify genes that were differentially expressed, we set a cutoff value of log2 (fold change) > 1 and *p* < 0.05.

### 5.13 Luciferase reporter assays

The putative ETS binding sites (CCTTCCTG) in the promoter sequence of GLI1 and CCL2 were obtained from JASPAR (http://jaspar.genereg.net/). To construct luciferase reporter plasmids,

GLI1 and CCL2 gene promoter regions were amplified from genomic DNA of HuCCT1 cells and cloned into PGL4.17 vector. Transfections with plasmids were performed with FectinMore Transfection Reagent, which was purchased from Chamot Biotechnology Co., Ltd. We used a dual luciferase assay (Promega) to measure luciferase activity. Data were normalized to renilla luciferase and are reported as means ± SEM for three separate experiments.

### 5.14 ChIP Assay

ChIP assays were performed using the Sonication ChIP Kit (Cell Signaling Technology). After the cells were cross‐linked with 1% formaldehyde, ultrasound fragmented them to produce fragmented DNA. The protein A/G magnetic beads used were purchased from Biolinkedin. Immunoprecipitations were carried out using anti‐EHF and an anti‐IgG as the control. Appropriate primers were used for real‐time PCR detection of immunoprecipitated DNA fragments. All assays were repeated three times. The primers used for ChIP are shown in Table S2.

### 5.15 Cytokine secretion assay

Cells were seeded in 10 cm plates (1 × 10^6^ cells/plate) with serum‐free RPMI medium for 24 h. The Conditioned medium (CM) was centrifuged for 20 min at 1000 × *g* at 4°C to remove particulates and then collected the supernatant to perform the assay. Human cytokine arrays (R&D, SYSTEMS) were incubated overnight with 1 mL of CM at 2−8°C on a rocking platform shaker. After washing with the buffer, the membrane was incubated in diluted Streptavidin HRP in a shaker at room temperature for 30 min. Specific immune complexes were detected using ECL Reagents (Thermo Fisher).

### 5.16 Co‐culture system

CCA cells and macrophages were co‐cultured in six‐well plates through 0.4‐μm‐transwell chambers. The macrophages were seeded in the upper layer and CCA cells were seeded in the lower layer. We added PMA (MedChemExpress) to the medium to induce THP‐1 to become macrophages, and IL‐4 and IL‐13(Novoprotein) to induce differentiation into M2‐type macrophages. The cells were cultured at 37°C and 5% CO_2_.

### 5.17 Flow cytometry

After anesthesia, tumor tissue was collected from mice and cut into small pieces of about 1 mm^3^, which were ground and filtered with a 70 μm filter to prepare single‐cell suspension. The primary antibody was incubated at room temperature for 45 min and analyzed by flow cytometry. Data were collected by Cytexpert software and analyzed by FlowJo software (BD Biosciences).

### 5.18 Statistical analysis

All values were presented as mean ± standard deviation. Each experiment was repeated three or more times as independent biological replicates. To determine statistical significance between two groups, Student's t‐tests (for normal distribution) or Wilcoxon signed‐rank tests (for matched pairs) were employed. For multiple groups, statistical analysis was performed using either one‐way ANOVA or two‐way ANOVA. Immunohistochemical score was analyzed using the chi‐squared test. Kaplan–Meier method was used to display the survival curves, and the statistical significance was assessed using the log‐rank test. Correlations were evaluated using a Pearson correlation test. Appropriate statistical analysis was performed across all figures, with *p* values < 0.05 considered statistically significant: *p < 0.05; **p < 0.01; ns, not significant. All statistical values were calculated using GraphPad Prism 8.0 software.

### 5.19 Bioinformatics analysis

To explore the clinical significance of EHF in human CCA tissues, we accessed the original data of TCGA‐CHOL (https://portal.gdc.cancer.gov/) and other four GEO dataset GSE26566, GSE119336, GSE107943, and GSE76297 dataset (https://www.ncbi.nlm.nih.gov/geo/). Subsequently, we used GraphPad to conduct Student's *t*‐tests (for normal distribution) or Wilcoxon signed‐rank tests (for matched pairs) to determine whether there was a statistical difference in EHF expression between cancerous tissues and paracancer tissue.

Analysis of the RNA‐SEQ results we generated, including identification of differentially expressed genes, as well as KEGG enrichment analysis, was provided by Wuhan Generead Biotechnology Co., Ltd.

## AUTHOR CONTRIBUTIONS

W.J.H. and H.F.L. were responsible for designing the experiments, while Y.M.L., Z.L., H.Z., J.L., and Z.L. carried out the experiments. C.S., Q.B.H., and H.W.Z. were responsible for collecting clinical samples and patient information. S.Q.H., D.A.R., and T.T.W. analyzed and interpreted the data. Y.M.L. wrote the paper, with guidance from X.P.C., Z.W.Z., and H.C. All authors read and approved the final manuscript.

## CONFLICT OF INTEREST STATEMENT

The authors declare no conflicts of interest.

## ETHICS STATEMENT

The study was approved by the ethics committee of Tongji Hospital, Tongji Medical College, Huazhong University of Science and Technology (Wuhan, China) and all human tissue samples were obtained with written informed consent from each patient (TJ‐IRB20201214). All animal experimental procedures were also approved by the Committee on the Ethics of Animal Experiments of Tongji Hospital (TJH‐202006006).

## Supporting information

Supporting Information

Supporting Information

## Data Availability

The data that support the findings of this study are available from the corresponding authors upon reasonable request. The raw sequence data have been deposited in Genome Sequence Archive in National Genomics Data Center, Beijing Institute of Genomics (https://ngdc.cncb.ac.cn/gsa‐human) with Project Accession No. PRJCA023813.
