# Supplementary material for: Transcription factor EHF drives cholangiocarcinoma development through transcriptional activation of glioma‐associated oncogene homolog 1 and chemokine CCL2
Source: MedComm (2020). 2024 May 13;5(5):e535. doi: 10.1002/mco2.535 (PMC11089446; doi:10.1002/mco2.535)
Supplement: Supplementary file 1 — Supporting Information [file MCO2-5-e535-s002.docx]

**Transcription factor EHF drives cholangiocarcinoma development through transcriptional activation of Glioma-associated oncogene homolog 1 and Chemokine CCL2**

**Yiming Luo^1^**^#^**, Zhi Li^2,5^**^#^**, He Zhu^1^, Junli Lu^1^,** **Zhen Lei^1^, Chen Su^1^, Furong Liu^1^, Hongwei Zhang^1^****, Qibo Huang****^1^, Shenqi Han^1^, Dean Rao^1^, Tiantian Wang^1^****, Xiaoping Chen^1,3,4^, Hong Cao^5^**^*^**, Zhiwei Zhang^1,3^**^*^**,** **Wenjie Huang^1,3^**^*^**, Huifang Liang^1,3^**^*^

**Supplementary Figure S1**
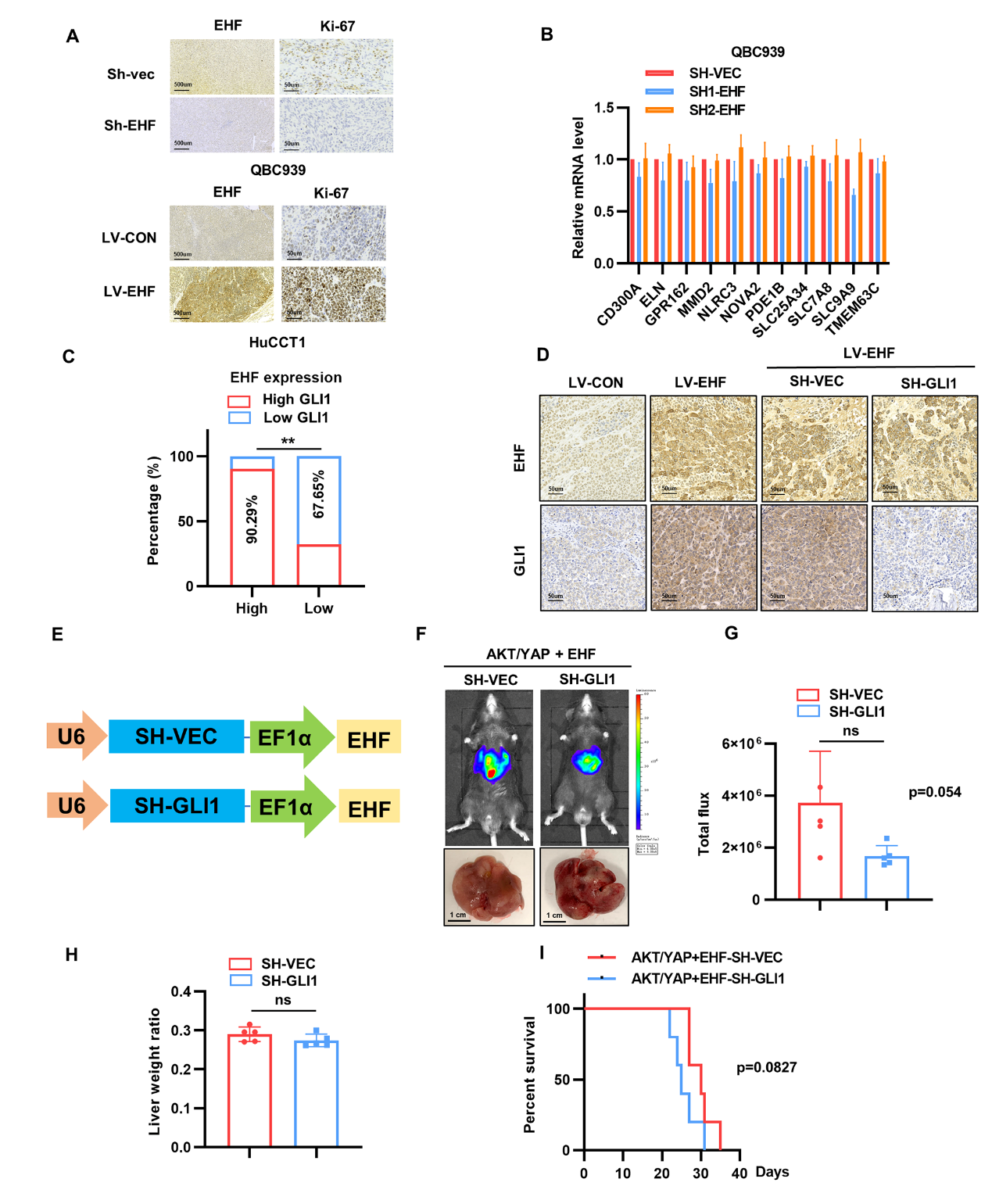


**Figure S1.** (A) Representative images of EHF and Ki-67 staining of the subcutaneous xenograft tumor. Scale bars (IHC), 50um. (B) The mRNA levels of 12 target genes in QBC939 cells. (C) The correlation between EHF and GLI1 in CCA samples. (D) Representative images of EHF and GLI1 staining of the orthotopic transplantation tumor. Scale bars (IHC), 50um. (E) Schematic diagram of constructing plasmid of SH-VEC and SH-EHF. (F-G) Representative bioluminescent pictures of AKT/Yap/EHF+ SH-VEC and SH-GLI1 mouse respectively. (H) The ratio of tumor weight to body weight. (I) Kaplan-Meier plots of the OS.

**Supplementary Figure S2**
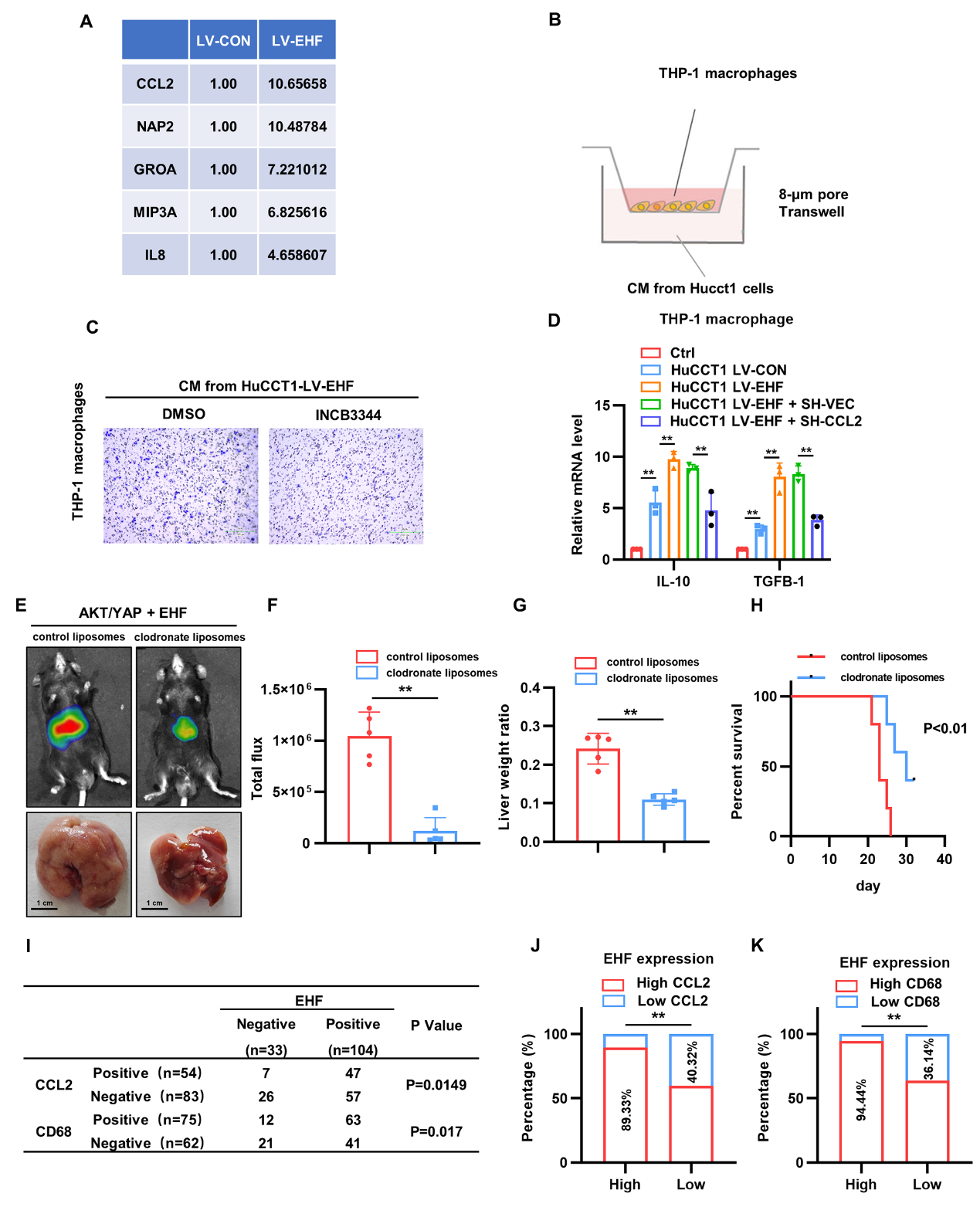
**Figure S2.** (A) The results of cytokine array. (B) Schematic diagram of Co-culture system**.** (C) Chemotaxis assays reflected the recruitment effect of CMs of HuCCT1 on THP1 macrophages. (D) The mRNA levels of IL-10 and TGFB-1 in THP-1 macrophages cultured with CMs of HuCCT1 cells. (E-F) Representative bioluminescent pictures of AKT/Yap/EHF+ liposomes and clodronate liposomes mouse respectively. (G) The ratio of tumor weight to body weight. (H) Kaplan-Meier plots of the OS. (I-K) The relevance of the expression of EHF and GLI1 in CCA cohorts.

**Supplementary Figure S3**
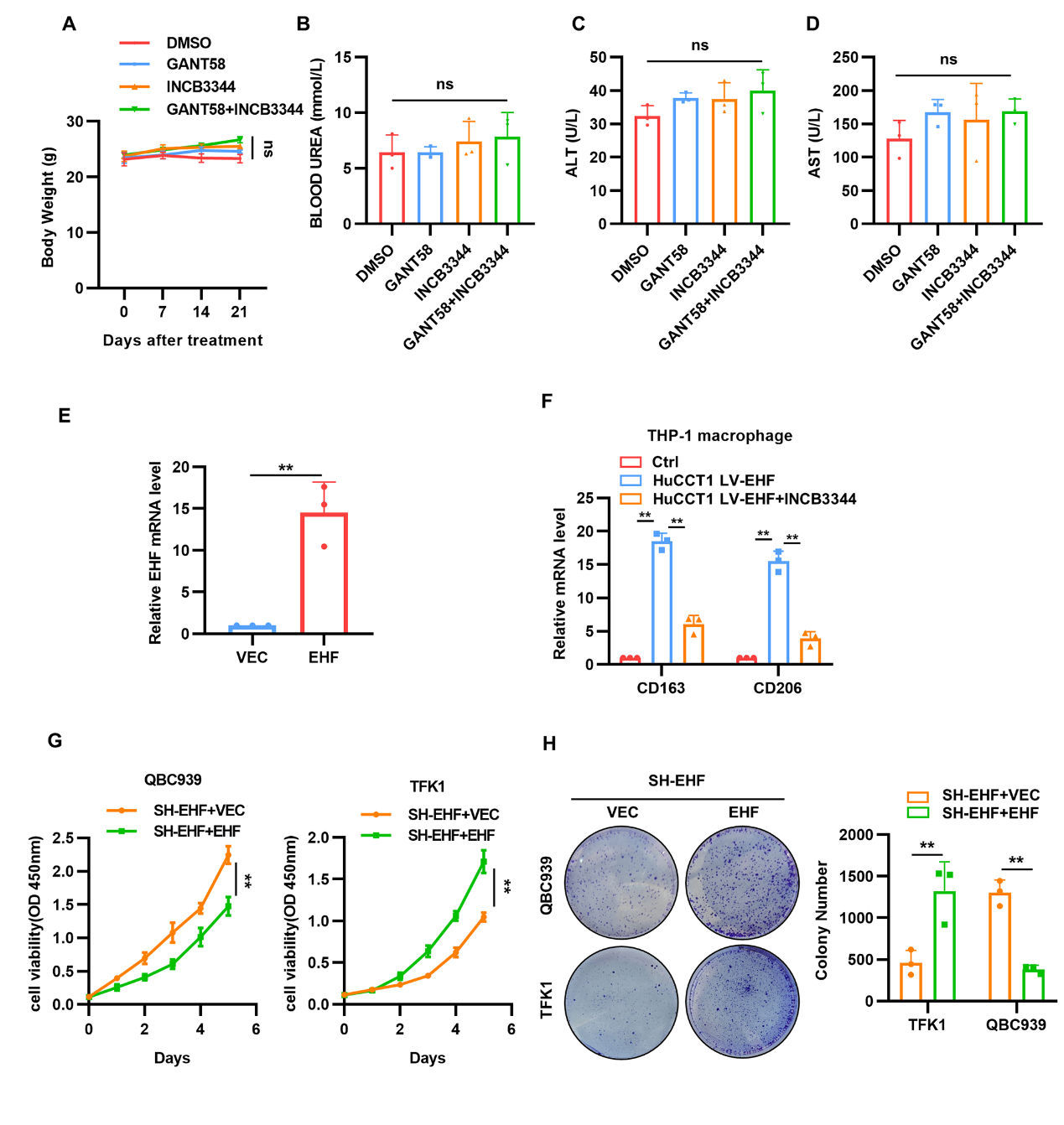


**Figure S3.** (A) The body weight of CCA mice in different group. (B-D) The blood urea, ALT and AST of different groups of mice. (E)The relative EHF mRNA level. (F) The mRNA levels of CD163 and CD206 in THP-1 macrophages cultured with CMs of HuCCT1 cells. (G) Growth curves measured by performing CCK-8 assay (OD 450nm). (H) Representative images of colony formation assays and colony counts.

| Antigen | Manufacturer | Catalog Number | Application |
| --- | --- | --- | --- |
| EHF | Proteintech | 27195-1-AP | 1:1000 for WB |
| EHF | Abcam | ab272671 | 1:100 for IHC |
| GAPDH | Proteintech | 60004-1-Ig | 1:10000 for WB |
| GLI1 | Proteintech | 66905-1-Ig | 1:1000 for WB  1:200 for IHC |
| CD68 | CST | 80119 | 1:100 for IHC |
| F4/80 | Abcam | ab111101 | 1:200 for IHC |
| KI-67 | Abcam | ab15580 | 1:200 for IHC |
| CK-19 | Abcam | ab52625 | 1:200 for IHC |
| CD45 | BD | 557659 | 1:1000 for Flow cytometry |
| F4/80 | BD | 743282 | 1:1000 for Flow cytometry |
| CD206 | Elabscience^®^ Biotechnology Co.,Ltd | E-AB-F1161E | 1:1000 for Flow cytometry |
| CD86 | BD | 740877 | 1:1000 for Flow cytometry |
| CD163 | HUABIO | ET1704-43 | 1:1000 for WB |
| β-actin | Proteintech | 81115-1-RR | 1:1000 for WB |
| CDK2 | Proteintech | 10122-1-AP | 1:1000 for WB |
| PCNA | Beijing Solarbio Science & Technology Co., Ltd. | K200030M | 1:2000 for WB |
| Goat Anti-Mouse IgG-Fc Secondary Antibody (HRP) | Sino Biological Inc. (Beijing, China) | SSA007 | 1:5000 for WB |
| Goat Anti-Rabbit IgG-Fc Secondary Antibody (HRP) | Sino Biological Inc. (Beijing, China) | SSA003 | 1:5000 for WB |

**Supplementary Table S1. Primary antibodies and secondary antibodies used in this study.**

**Supplementary Table S2. Primer sequences and shRNAs used in this study.**

| **Primers for RT-qPCR** | | |
| --- | --- | --- |
| Gene | Forward primer (5'-3') | Reverse primer (5'-3') |
| GAPDH | GATGGGTGTGAACCACGAGAA | GGGCCATCCACAGTCTTCTG |
| EHF | TGCAGCATCTGAAGTGGAAC | AGGAAGGTGACTGGTGGTTG |
| GLI1 | CAGCCAGATGCAATCAATGCC | TGGAATCCTGAACCCACTTCT |
| CCL2 | AGCGTGAGCCTGAATCTGTG | CAGCATGTACTGGGCTTTGAA |
| RT-CD163 | TTTGTCAACTTGAGTCCCTTCAC | TCCCGCTACACTTGTTTTCAC |
| RT-ARG1 | TGGACAGACTAGGAATTGGCA | CCAGTCCGTCAACATCAAAACT |
| RT-IL10 | GACTTTAAGGGTTACCTGGGTTG | TCACATGCGCCTTGATGTCTG |
| RT-CD206 | GGGTTGCTATCACTCTCTATGC | TTTCTTGTCTGTTGCCGTAGTT |
| RT-TGFB1 | GGCCAGATCCTGTCCAAGC | GTGGGTTTCCACCATTAGCAC |
| RT-Cd300a | CATCAACGTCAATGACACCTGC | CACCCACTGCAAACAGGGTA |
| RT-Eln | GCAGGAGTTAAGCCCAAGG | TGTAGGGCAGTCCATAGCCA |
| RT-GPR162 | GTGACGACTATGCAGAGGGC | AAGTAGTGGACTCTCTCAAGAGG |
| RT-MMD2 | CAGAAGACGAAATACGCGAGG | GCCGCATGTTCATACTCTGTG |
| RT-Nlrc3 | CTGGATAGGACACCGGATGC | TGAAGTCGTGTTCCCTCAGC |
| RT-NOVA2 | AAGGCGAATACTTCCTGAAGGT | TACTAGGCATACCCGCTCTGT |
| RT-PDE1B | CTGCGCTACATGGTGAAGCA | CAAGATTTGCCGTGTCTCATCTA |
| RT-SLC25A34 | ATGGCGTTCGTTTCTACTGCT | CAGGACAGTCTGGTGATTGTG |
| RT-SLC7A8 | AGGCTGGAACTTTCTGAATTACG | ACATAAGCGACATTGGCAAAGA |
| RT-SLC9A9 | GTGGAGCTGCTTGTCTTCAAT | GCAGAGTTGATGGACTGAAAGTT |
| RT-TMEM63C | CCATCAGGTTCCAGTGTGTG | ACGTTCATCATCCACGCATA |
| **Primers for shRNA** | | |
| sh-vec | TTCTCCGAACGTGTCACGT | |
| sh1-EHF | ACCCAAGGTTGGCTATAAT | |
| sh2-EHF | GTGCTCTTCATCAACTAAT | |
| sh1-Ehf | GCTCGTGGATGGAGAGA | |
| sh2-Ehf | GCTCAGATCTCCATGAC | |
| sh-GLI1 | CCTGTGTACCACATGACTCTA | |
| sh-Gli1 | GCATGGGAACAGAAGGACTTT | |
| si-Ccl2 | GCAAGATGATCCCAATGAGTA | |
| **Primers for Chip** | | |
| Gene | Forward primer (5'-3') | Reverse primer (5'-3') |
| GLI1 | CTTCCCTGTCAGGCAGTACC | CCGTTCTCACACATGACACC |
| CCL2 | TGTTTACACAATCCTACAGTTCTGC | TTACCTTCAGGCCACATTCC |

**Supplementary Table S3. Correlation between clinicopathological features and EHF expression in 137 CCA cases**

| **Clinicopathological variables** | **Tumor EHF expression** | | ***P***  ***Value*** |
| --- | --- | --- | --- |
|  | **Negative**  **(n=28)** | **Postive**  **(n=95)** |  |
| ***Gender*** |  |  |  |
| Male | 14 | 57 |  |
| Female | 14 | 38 | 0.3884 |
| ***Age*** |  |  |  |
| <60 years | 19 | 72 |  |
| >60 years | 9 | 23 | 0.4638 |
| ***Tumor size**** |  |  |  |
| <5 cm | 16 | 31 |  |
| >5 cm | 12 | 64 | **0.0265** |
| ***Tumor number*** |  |  |  |
| Single | 18 | 75 |  |
| Multiple | 10 | 20 | 0.135 |
| ***Cirrhosis*** |  |  |  |
| Absent | 17 | 59 |  |
| Present | 11 | 35 | 0.8285 |
| ***Tumor encapsulation*** |  |  |  |
| Absent | 24 | 82 |  |
| Present | 4 | 13 | 1 |
| ***TNM stage**** |  |  |  |
| I-II | 18 | 39 |  |
| III | 10 | 56 | **0.0337** |
| ***Lymphatic Invasion*** |  |  |  |
| Absent | 21 | 68 |  |
| Present | 7 | 27 | 0.8133 |
| ***Microvascular Invasion*** |  |  |  |
| Absent | 23 | 72 |  |
| Present | 5 | 23 | 0.6115 |
| ***Differentiation grade*** |  |  |  |
| High grade | 13 | 42 |  |
| Low grade | 15 | 53 | 0.8328 |
