# Supplementary material for: Transcription factor EHF drives cholangiocarcinoma development through transcriptional activation of glioma‐associated oncogene homolog 1 and chemokine CCL2
Source: MedComm (2020). 2024 May 13;5(5):e535. doi: 10.1002/mco2.535 (PMC11089446; doi:10.1002/mco2.535)
Supplement: Supplementary file 2 — Supporting Information [file MCO2-5-e535-s001.docx]

**Figure S1.** (A) Representative images of EHF and Ki-67 staining of the subcutaneous xenograft tumor. Scale bars (IHC), 50um. (B) The mRNA levels of 12 target genes in QBC939 cells. (C) The correlation between EHF and GLI1 in CCA samples. (D) Representative images of EHF and GLI1 staining of the orthotopic transplantation tumor. Scale bars (IHC), 50um. (E) Schematic diagram of constructing plasmid of SH-VEC and SH-EHF. (F-G) Representative bioluminescent pictures of AKT/Yap/EHF+ SH-VEC and SH-GLI1 mouse respectively. (H) The ratio of tumor weight to body weight. (I) Kaplan-Meier plots of the OS.

**Figure S2.** (A) The results of cytokine array. (B) Schematic diagram of Co-culture system**.** (C) Chemotaxis assays reflected the recruitment effect of CMs of HuCCT1 on THP1 macrophages. (D) The mRNA levels of IL-10 and TGFB-1 in THP-1 macrophages cultured with CMs of HuCCT1 cells. (E-F) Representative bioluminescent pictures of AKT/Yap/EHF+ liposomes and clodronate liposomes mouse respectively. (G) The ratio of tumor weight to body weight. (H) Kaplan-Meier plots of the OS. (I-K) The relevance of the expression of EHF and GLI1 in CCA cohorts.

**Figure S3.** (A) The body weight of CCA mice in different group. (B-D) The blood urea, ALT and AST of different groups of mice. (E)The relative EHF mRNA level. (F) The mRNA levels of CD163 and CD206 in THP-1 macrophages cultured with CMs of HuCCT1 cells. (G) Growth curves measured by performing CCK-8 assay (OD 450nm). (H) Representative images of colony formation assays and colony counts.
